# Two new millipede genera and species from Kangaroo Island, South Australia (Polydesmida, Dalodesmidae, Spirostreptida, Cambalidae)

**DOI:** 10.3897/zookeys.1295.214938

**Published:** 2026-10-23

**Authors:** Robert Mesibov

**Affiliations:** 1 Ulverstone, Tasmania 7315, Australia Ulverstone Tasmania Australia

**Keywords:** Endemic, millipede, morphology, taxonomy

## Abstract

*Colobothrix deckeri***gen. et sp. nov**. (Polydesmida, Dalodesmidae) has sphaerotrichomes with very short, blunt setae and long, thin telopodites divided at the tips into short processes. *Notonesocambala adoceta***gen. et sp. nov**. (Spirostreptida, Cambalidae) has a large spine and an expanded, lamellar apex with anteriorly directed tab on the coxite process of the anterior gonopod. The two new species have recently been collected in a small part of the northwestern portion of Kangaroo Island, South Australia. *Colobothrix deckeri***gen. et sp. nov**. was also collected in 1936 on the lower Yorke Peninsula, ca 50 km distant on the South Australian mainland, but has not been found there more recently.

## Introduction

In Australia, polydesmidan millipedes in the family Dalodesmidae have only been described from higher-rainfall areas in the east and southwest of the continent, leaving a distribution gap more than 2000 km wide between western Victoria and southwest Western Australia. Most of the southern half of Australia within this gap has an annual rainfall less than 400 mm. The wettest portion is in South Australia’s Adelaide Hills, parts of which have an annual rainfall of ca 1110 mm (Bureau of Meteorology 2026). However, the only native Polydesmida found in the Hills so far have been Haplodesmidae (Mesibov 2013) and Paradoxosomatidae (Decker 2016).

The “dalodesmid gap” is here reduced with the description of a new species from South Australia’s Kangaroo Island and the lower Yorke Peninsula. A new species of Cambalidae (Spirostreptida) from within the Kangaroo Island range of the new dalodesmid is also described.

## Materials and methods

All specimens are stored in ethanol in the South Australian Museum (SAMA) or the Western Australian Museum (WAM). For future use as DNA vouchers, specimens of the two new species were transferred to 100% ethanol within one week after capture and preservation in 75% ethanol.

Colour images were acquired with a Leica K5C camera on a Leica M205C stereo microscope and stacked with LAS X v5.3.3 software. Gonopods were cleared in 90+% lactic acid, temporarily mounted in a 1:1 glycerol:water mixture and imaged with an Omax A35180U3 eyepiece video camera mounted on an AmScope B210 microscope, with subsequent focus-stacking of manually focused images using Zerene Stacker 1.04 software. Image backgrounds have been edited to remove distracting highlights and artefacts. Scanning electron microscope images were acquired using a Hitachi SU-70; body parts were examined after air-drying and sputter-coating with platinum. The distribution maps were prepared with QGIS 3.40 and figures and plates composed with GIMP 3.04.

Locality details are given with latitude and longitude in decimal degrees based on the WGS84 datum. The estimated uncertainty for a locality is the radius of a circle around the given position.

## Results

### Order Polydesmida Pocock, 1887

**Suborder Dalodesmidea Hoffman, 1980**

**Family Dalodesmidae Cook, 1896**

#### Colobothrix

Taxon classificationAnimaliaPolydesmida

Mesibov gen. nov.

B20D4DE1-3668-5C65-AB83-8364A9C76342

https://zoobank.org/F7AB51DA-4DE4-49F5-979D-949221A38A5C

##### Type species.

*Colobothrix deckeri* Mesibov, sp. nov., by monotypy and present designation.

##### Other assigned species.

None.

##### Diagnosis.

Three other described Australasian dalodesmid genera with long, thin, straight, closely appressed telopodites divided at the tips are *Noteremus* Mesibov, 2009 and *Setoisenoton* Mesibov, 2010 from Tasmania and *Polydactylogonus* Mesibov, 2025 from alpine eastern Victoria. The two *Noteremus* species (Mesibov 2009) have clusters of closely packed, rod-like structures at the telopodite tips that are absent in *Colobothrix* gen. nov., and *Noteremus* spp. also lack sphaerotrichomes on male legs. In *Colobothrix* gen. nov. the posterior telopodite process is thickened, distally expanded and directed posteriorly, the solenomere is directed laterally and sphaerotrichome setae are short and blunt, while in *Setoisenoton* the posterior telopodite process is flat, narrow and directed distally, the solenomere is directed distally and sphaerotrichome setae are long and pointed (Mesibov 2010). There are two apical processes in *Colobothrix* gen. nov. and five in *Polydactylogonus*, which has a long, thin, pointed seta on each sphaerotrichome (Mesibov 2025).

##### Description.

As for *Colobothrix deckeri* sp. nov.

##### Name.

Greek *kolobos* (stunted) + *thrix* (hair), for the stunted setal shafts on the sphaerotrichomes; gender feminine.

##### Remarks.

Previously noted variations in the setal shaft portion of dalodesmid sphaerotrichomes are “gently tapered”, “sharply pointed”, “slightly expanded at the tip” and “wholly absent” (Mesibov 2004), to which I can now add “reduced to a short, blunt process”. The function of polydesmidan sphaerotrichomes is currently unknown.

#### Colobothrix deckeri

Taxon classificationAnimaliaPolydesmida

Mesibov sp. nov.

68628FBD-02DD-5F15-A192-04C194E8754E

https://zoobank.org/8BFD38AA-8B27-42CC-9E77-7EACA7DC39AD

Figs 1A, 2, 3

##### Type material.

***Holotype***. Male, South Australia • Kangaroo Island, Snug Cove Road, -35.7708, 136.8809 ±25 m, 280 m a.s.l., 2026-08-06, leg. Robert Mesibov, under rotting wood in low forest E of road, SAMA OM-2218. ***Paratypes***. • 1 female, details as for holotype, SAMA OM-2219; 3 males, 1 female, Kangaroo Island, De Mole Estate, -35.7330, 136.8087 ±25 m, 280 m a.s.l., 2021-05-23, leg. Jessica Marsh, site 6, open sugar gum near creek, field number M7, SAMA OM-2224–OM-2227.

##### Additional material.

• 4 juveniles (in 100% EtOH), Kangaroo Island, Snug Cove Road, -35.7699, 136.8803 ±25 m, 290 m a.s.l., 2026-08-05, leg. Robert Mesibov and Richard Glatz, native woodland remnant in *Eucalyptus globulus* plantation, in well-rotted litter in surface depression, SAMA OM-2259–OM-2262; • 8 juveniles, same details but -35.7698, 136.8807, 2026-08-06, leg. Robert Mesibov, SAMA OM-2263–OM-2270; • 1 female, Kangaroo Island, De Mole Estate, -35.7306, 136.8229 ±25 m, 110 m a.s.l., 2021-05-21, leg. Jessica Marsh, Site 1, open sugar gum, litter beaten from understorey vegetation, SAMA OM-2272; • 1 juvenile, same details but -35.7182, 136.8511, 120 m a.s.l., 2021-07-29, field number M1, SAMA OM-2273; • 4 females, same details but -35.7311, 136.8068, 100 m a.s.l., 2021-05-22, assassin spider habitat, on ground or under log on ground, open sugar gum near creekline, field number M3, SAMA OM-2293–OM-2296; • 2 males, 6 females, Kangaroo Island, Waterfall Creek Trail, near waterfall, -35.6956, 136.9103 ±25 m, 130 m a.s.l., 2010-05-09, leg. Michael G. Rix and Danilo Harms, sifting/beating, field number ArPTC#77, WAM ARACH:112629; • 1 female, same details, WAM ARACH:112630; • 1 male in fragments, South Australia, Yorke Peninsula, near Marion Bay, -35.2452, 136.9720 ±10 km, 1936-04-09, leg. Herbert Womersley, SAMA OM-491.

##### Description.

Male (Fig. 1A) and female with head + 19 rings. Male/female overall length ca 19/20 mm, maximum midbody vertical diameter ca 1.4/1.6 mm. Colour yellowish-brown with reddish-brown speckling; speckling denser dorsally, concentrated at tergal and metatergal margins, around ozopores and distally on antennae.

**Figure 1.**
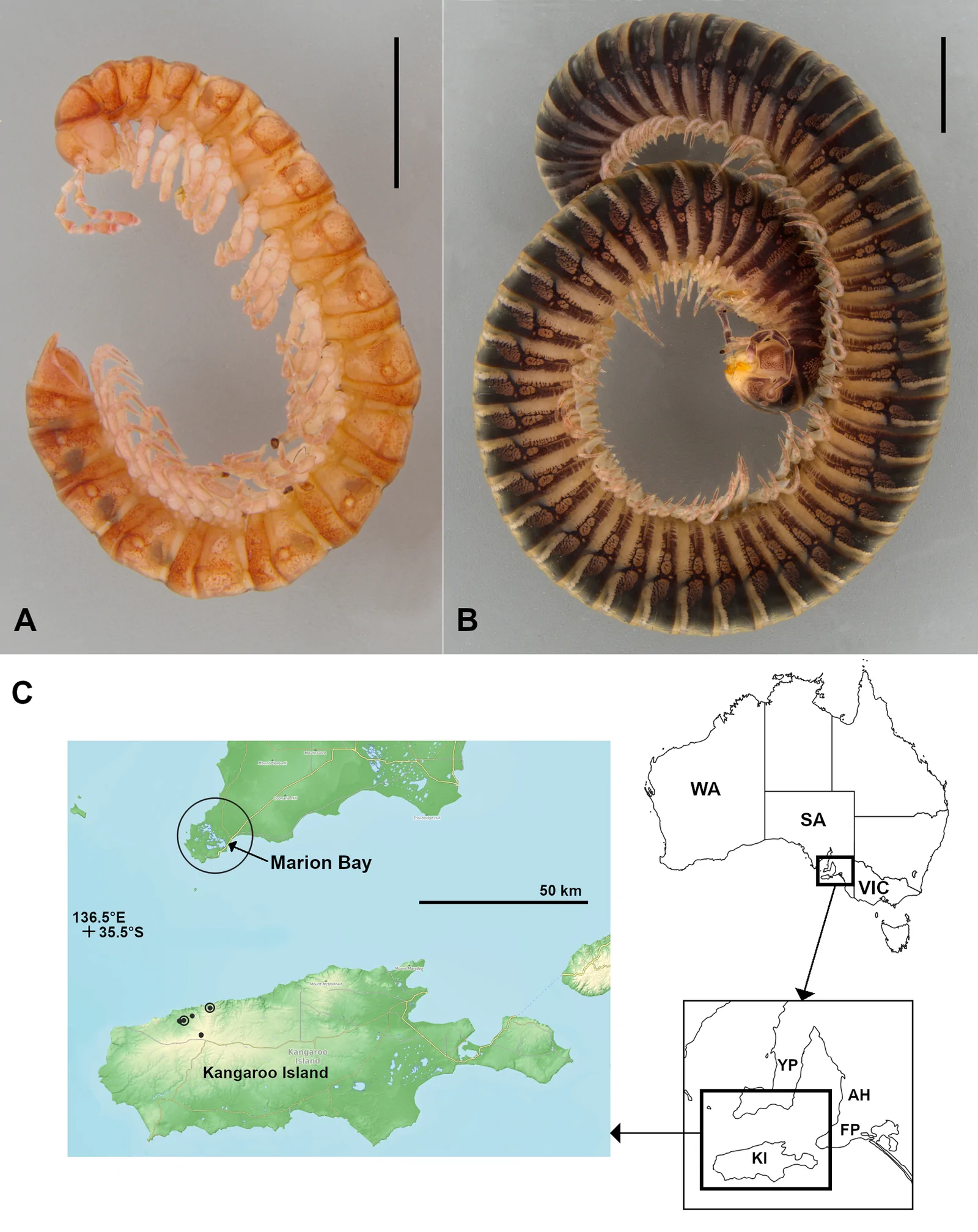
**A, B**. Male holotypes, original images by Ben Parslowe. **A**. *Colobothrix deckeri* gen. nov., sp. nov., SAMA OM-2218; **B**. *Notonesocambala adoceta* gen. nov., sp. nov., SAMA OM-2220; **C**. Localities map for *C. deckeri* gen. nov., sp. nov. (black dots) and *N. adoceta* gen. nov., sp. nov. (small circles) on Kangaroo Island. Large circle near Marion Bay on Yorke Peninsula marks area in which *C. deckeri* gen. nov., sp. nov. was collected in 1936. Index maps show other areas mentioned in text; abbreviations: **AH** Adelaide Hills, **FP** Fleurieu Peninsula, **KI** Kangaroo Island, **SA** South Australia, **VIC** Victoria, **WA** Western Australia, **YP** Yorke Peninsula. Localities base map from OpenTopoMap project (https://opentopomap.org). Scale bars: 2.5 mm (**A, B**).

Male with a few setae on vertex, frons sparsely setose with long setae, clypeus more densely setose. Vertigial sulcus evident, extending almost to level of antennal sockets. Antennae clavate, slender, reaching tergite 2 when manipulated dorsally. Relative antennomere lengths (2=3)>(4=6)>5; antennomere 7 ca 1/4 length of antennomere 6; antennomere 6 widest. Antennal sockets separated by ca 2× socket diameter.

Collum reniform in dorsal view, the corners rounded. Tergite 2 with paranotum reaching below level of collum and tergite 3, the margin thickened and slightly produced laterally; no ventral pit. Paranotal margins similar but smaller on tergites 3 and 4. Tergite widths (head=5)>(collum=4)> 3>2; widths 6-16 subequal, 17 and 18 narrower. Tergite 3 shorter than tergites 2 and 4.

Diplosegments with pronounced waist; prozonite and metazonite smooth with no sculpturing; no metazonital setae. Paranotum a little above 1/2 ring height, greatly reduced, margin thickened and slightly convex laterally; posterior corner slightly produced and rounded on most rings. Limbus (Fig. 2B) a single row of thin, flat, multi-pointed projections. Midbody sternites a little wider than long, transverse impression deeper than longitudinal impression. Pore formula normal; ozopores small, set on slight swellings at rear of paranota, opening dorsolaterally. Spiracles small, round with slightly thickened rims; on diplosegments with anterior spiracle over anterior leg and posterior spiracle midway between leg bases. Epiproct with truncate tip extending past anal valves; spinnerets in square array with ventral pair in shallow recess in epiproct tip. Hypoproct paraboloid.

**Figure 2.**
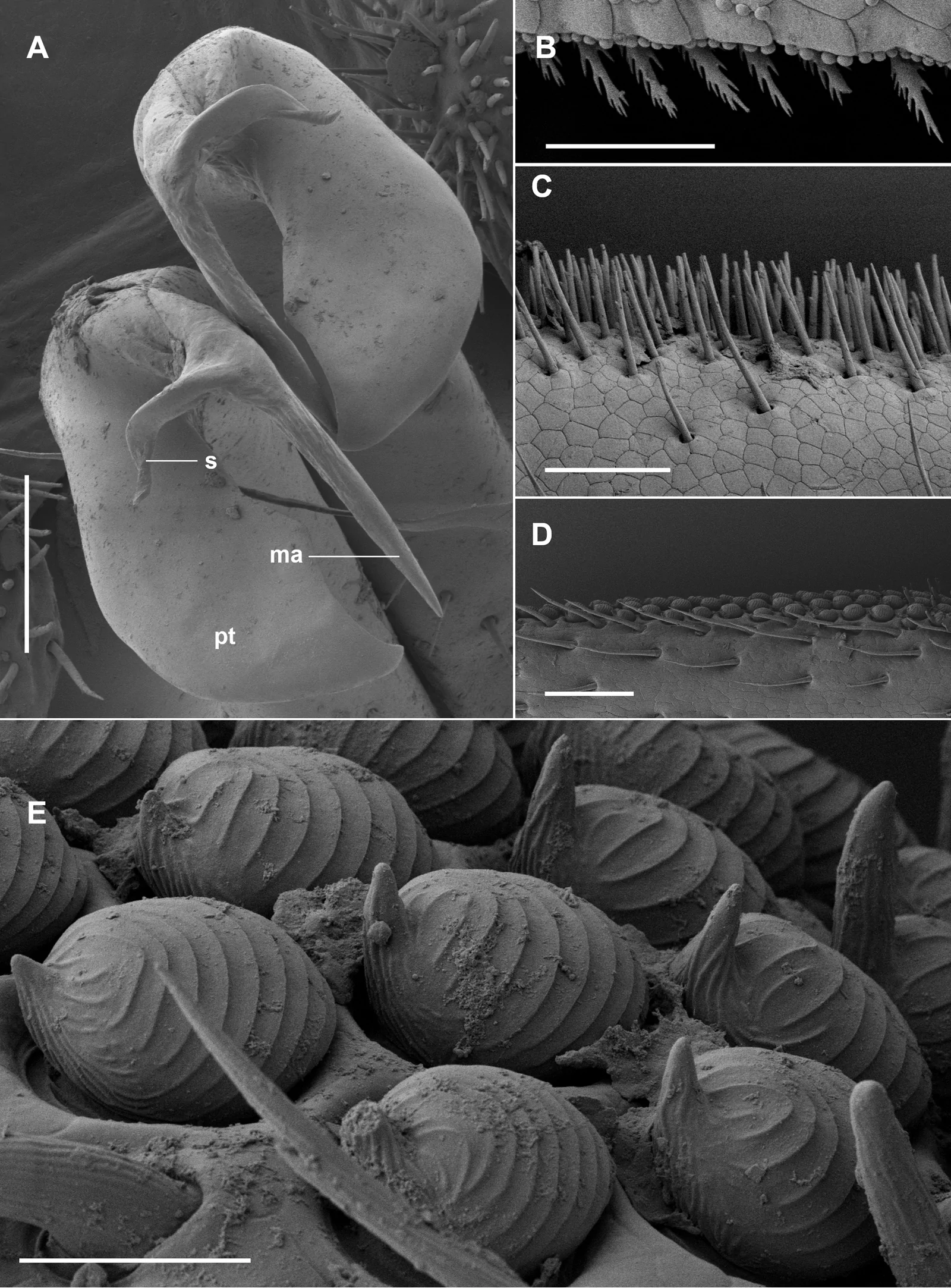
*Colobothrix deckeri* gen. nov., sp. nov., male paratypes ex SAMA OM-2224–OM-2227. **A**. Tips of gonopod telopodites; abbreviations: **ma** medial arm of apical “T”, **pt** posterior tab, **s** solenomere; **B**. Midbody ring limbus, detail; **C**. Brush setae on leg 6 prefemur, distal to left; **D**. Sphaerotrichomes on leg 6 tarsus, distal to left. **E**. Sphaerotrichomes on leg 6 tarsus, detail, distal to left. Scale bars: 0.1 mm (**A**); 0.025 mm (**B**); 0.05 mm (**C**, **D**); 0.01 mm (**E**).

Anterior legs thickened with prefemur greatly swollen dorsally, femur less so. Midbody legs ca 1.3× ring diameter; podomere lengths tarsus>femur>prefemur>(tibia=tarsus); tarsus straight; claw ca 1/6 of tarsus length. Brush setae and sphaerotrichomes present on all but most posterior legs. Brush setae (Fig. 2C) long, slightly tapering with truncate tips, densely clustered on prefemur, femur and postfemur, on anterior legs also on coxa. Sphaerotrichomes (Fig. 2D) on tibia and tarsus; sphaerotrichome base (Fig. 2E) ovoid, transversely ridged, slightly embedded in podomere surface; very short seta arising from distal end of base, 1/4 of base length but length somewhat variable, conical with bluntly rounded tip directed ventrally or ventrodistally.

Rings 5 and 6 with sternites excavate to accommodate retracted gonopod telopodites; long sternal setae medially on ring 6. Gonopore small, round, at distomedial end of leg 2 coxa. Aperture ovoid with posterior and posterolateral margin a little extended, ca 1/2 width of ring 7 prozonite.

Gonocoxa small, subconical, sparsely setose; cannula prominent. Telopodites (Figs 2A, 3) slender, straight, not tapering, just reaching leg 5 bases when retracted, medially appressed but not fused, bent slightly anteriorly at ca 1/4 telopodite height, sparsely setose at base. Telopodite at ca 7/8 height with thick, tab-like, posteriorly directed process with posteromedial margin rounded and deeply excavate. Telopodite apex T-shaped (Fig. 2A); medial arm of “T” short, tapering to sharp point, directed posterobasally; lateral arm (= solenomere), short, flattened, directed distolaterally and curving basally, apex twisted. Prostatic groove (Fig. 3B) running from medial to anteromedial surface of telopodite before turning abruptly laterally to enter solenomere.

**Figure 3.**
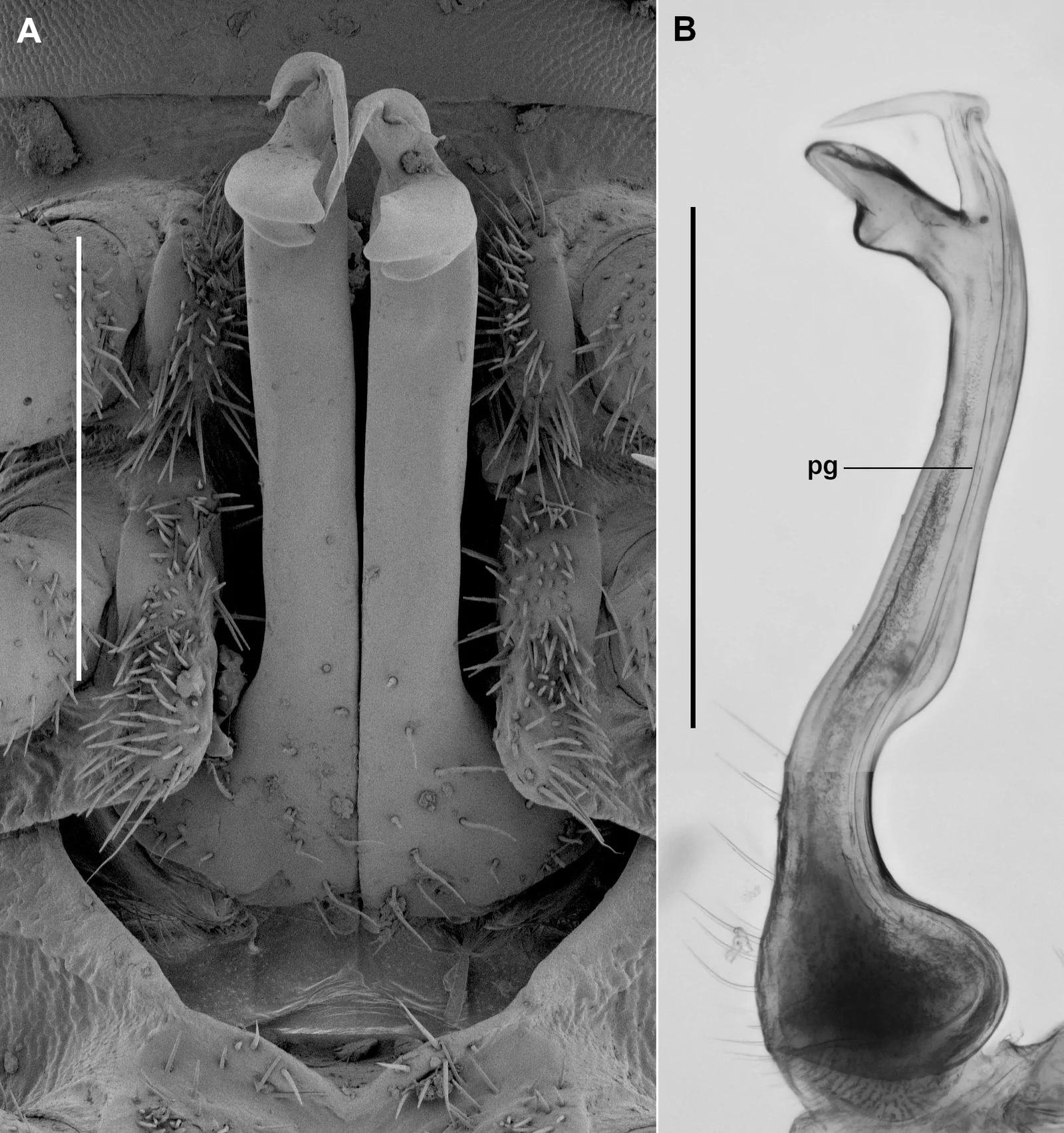
*Colobothrix deckeri* gen. nov., sp. nov., male paratypes ex SAMA OM-2224–OM-2227. **A**. Ventral view of gonopod telopodites in situ; **B**. Medial view of cleared right gonopod telopodite; abbreviation: **pg** prostatic groove. Scale bars: 0.5 mm.

Female larger than male and with thinner legs; epigynum narrow, subrectangular; cyphopods not examined.

##### Name.

Noun in the genitive case. For my friend and colleague Peter Decker, who identified the 1936 specimen as a dalodesmid and recognised its significance (see Remarks).

##### Distribution.

Known from five sites within a range envelope of ca 60 km^2^ in the northwestern portion of Kangaroo Island, and from one disjunct site at the southern end of the Yorke Peninsula, South Australia (Fig. 1C).

##### Remarks.

No strong smell of defensive secretion was detected in living or preserved specimens. The lack of a strong-smelling defensive secretion in *C. deckeri* gen. nov., sp. nov. suggests that any secretion is only weakly cyanogenic, rather than phenolic or based on vinyl ketones as in some other dalodesmids (Jones et al. 2018).

As explained in the following chronology, the “shelf life” (Fontaine et al. 2012) of *Colobothrix deckeri* gen. nov., sp. nov. between first known collection and description has been 90 years.

**1936**: The entomologist Herbert Womersley collected a male polydesmidan on South Australia’s lower Yorke Peninsula during a little-known scientific expedition. I have been unable to locate a report from the expedition, but expedition personnel are listed in a contemporary newspaper article (Anonymous 1936) which names Marion Bay as the site of the expedition’s base camp. SAMA labels for the millipede and for two other Womersley specimens (buprestid beetles) give the collecting date as “9 Apr. 1936”, but this may have been just the starting date of the expedition. Womersley (1937, p. 98) wrote that he collected a silverfish “from under fallen logs at Inneston, Yorke Peninsula, South Australia, in April, 1936”; Inneston is ca 8 km from Marion Bay.

**2010**: Two males and seven females of Womersley’s millipede were collected on Kangaroo Island, ca 50 km south of Marion Bay, by arachnologists Danilo Harms and Michael Rix. The specimens were by-catch from a spider search (see Discussion) and were deposited in the Western Australian Museum (WAM). They were subsequently identified as Dalodesmidae by WAM-based myriapodologist Catherine Car.

**2015**: Peter Decker, working then at Senckenberg Museum für Naturkunde Görlitz (Germany), found fragments of Womersley’s 1936 male in a SAMA loan of Paradoxosomatidae. He sent me photomicrographs and SEMs of the fragments.

**2016 and 2017**: In the austral winters of these two years I searched for Womersley’s millipede in forest, woodland and scrub within ca 20 km from Marion Bay on the lower Yorke Peninsula. The searches were unsuccessful, but the area yielded a new paradoxosomatid species (Mesibov 2017).

**2021**: Arachnologist Jessica Marsh collected Womersley’s millipede during a targeted spider survey (see Discussion) at sites several km from the 2010 Harms-Rix site.

**2026**: While looking online for southern Australian occurrences of Dalodesmidae I found the WAM records of the Harms-Rix specimens. I requested photos of one of the males from WAM curator Mark Harvey, who kindly provided high-quality photomicrographs of a male indistinguishable from the SAMA specimen in somatic details and gonopod structure.

In the austral winter of 2026 I searched for the new dalodesmid at the 2010 Harms-Rix locality but failed to find it. However, I collected fresh material at two nearby sites and identified Womersley’s millipede among the litter invertebrates collected in 2021 by Jessica Marsh, who kindly allowed me to deposit the specimens in SAMA.

### Order Spirostreptida Brandt, 1833

**Family Cambalidae Bollman, 1893**

#### Notonesocambala

Taxon classificationAnimaliaSpirostreptida

Mesibov gen. nov.

CF954CD2-0982-5325-A108-3BC5667EE65C

https://zoobank.org/57D916FE-0D2B-4B9A-8929-B374AFF38A73

##### Type species.

*Notonesocambala adoceta* Mesibov, sp. nov., by monotypy and present designation.

##### Other assigned species.

None.

##### Diagnosis.

Distinguished by the structure of the anterior gonopod. Differs from species in the seven described Australasian cambalid genera *Apocoptogonus* Jeekel, 2006, *Dimerogonus* Attems, 1903, *Eumastigonus* Chamberlin, 1920, *Euryischiogonus* Jeekel, 2009, *Proscelomerion* Verhoeff, 1924, *Stenischiogonus* Jeekel, 2009 and *Tasmanocambala* Mesibov, 2019 in having a wide lamellar apex, an anteriorly directed tab and a spine-like anteromedial projection on the coxal process. Differs from *Dimerogonus*, *Euryischiogonus* and *Stenischiogonus* in having the telopodite longer than the coxal process.

##### Description.

As for *Notonesocambala adoceta* sp. nov.

##### Name.

Greek *notos* (south) + *nesos* (island) + *Cambala* (type genus of Cambalidae); gender feminine. For Kangaroo Island, the southern Australian island where the genus is found.

##### Remarks.

As noted previously (Mesibov 2019) the classification of millipedes in the suborder Cambalidea is unsettled. For practical purposes I group the Australasian cambalideans into Cambalidae, with an anterior gonopod flagellum and relatively well-developed posterior gonopods, and Iulomorphidae, without a flagellum and with greatly reduced posterior gonopods. *Notonesocambala* gen. nov. clearly belongs in the first group.

#### Notonesocambala adoceta

Taxon classificationAnimaliaSpirostreptida

Mesibov sp. nov.

4DBE1428-7D6A-5AC2-B6AF-04CC408AFDF8

https://zoobank.org/F5DA54C9-92D9-4CB5-AFB6-1385CDD2AC63

Figs 1B, 4–6

##### Type material.

***Holotype***. Male, South Australia • Kangaroo Island, -35.6957, 136.9101 ±25 m, 130 m a.s.l., 2026-08-05, leg. Robert Mesibov and Richard Glatz, near first creek crossing on track to Billy Goat Falls, SAMA OM-2220. ***Paratypes***. • 1 male, details as for holotype, SAMA OM-2221; 1 male in 100% EtOH, details as for holotype, OM-2222; 1 male, South Australia, Kangaroo Island, De Mole Estate, -35.7306, 136.8229 ±25 m, 110 m a.s.l., 2021-05-21, leg. Jessica Marsh, Site 1, open sugar gum, litter beaten from understorey vegetation, SAMA OM-2223.

##### Additional material.

None.

##### Description.

Male (Fig. 1B) dark gray with pale dorsolateral mottling, prozonite pale on most rings. Trunk with up to 62 podous rings, midbody vertical diameter to ca 3 mm, overall length to ca 50 mm.

Head smooth, vertigial sulcus pale and distinct. Ommatidia in 4–5 rows, posterior row longest with up to 13 ocelli. Antennal sockets separated by ca 3× socket diameter. Antennae slender, reaching tergite 3 when manipulated dorsally; antennomere lengths (2=3)>4>(5=6); antennomere 6 not wider than distal end of antennomere 5; antennomere 7 ca 1/4 length of antennomere 6; 4 apical cones. Gnathochilarial stipetes separated by wide, rounded mentum (Fig. 4A); promentum triangular with convex posterior margin. Ventral margin of mandibular stipes slightly recurved outwards, forming narrow shelf. Collum subelliptical in dorsal view, corners slightly rounded; collum ca 3× length of tergite 2. Trunk rings smooth, shiny; suture visible both dorsally and ventrally; no prozonite striae; metazonite striae horizontal, terminating at suture. Limbus short, lamellar. Ozopores small, beginning ring 6, at ca 1/2 ring height, halfway between suture and posterior metazonite margin. Telson with dorsal margin of preanal ring very slightly produced, not forming distinct epiproct; hypoproct short, broadly paraboloid dorsally.

**Figure 4.**
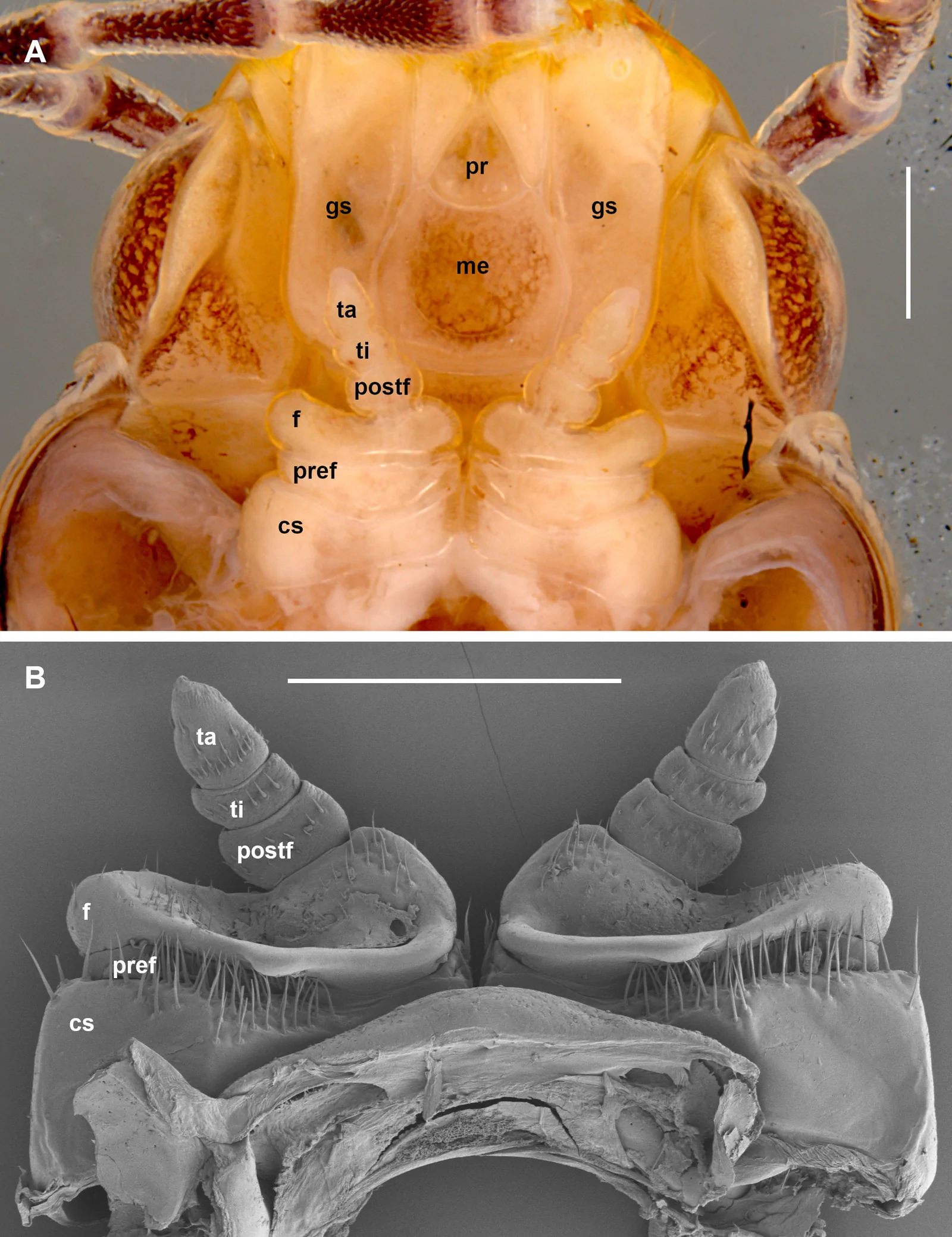
*Notonesocambala adoceta* gen. nov., sp. nov., male paratype, SAMA OM-2221. **A**. Posterior view of head and legpair 1; **B**. Anterior view of legpair 1. Abbreviations: **cs** coxosternite, **f** femur, **gs** gnathochilarial stipe, **me** mentum, **postf** postfemur, **pr** promentum, **pref** prefemur, **ta** tarsus, **ti** tibia. Scale bars: 0.5 mm.

Legpair 1 (Fig. 4) with broad coxosternite bearing long setae anteriorly; prefemur and femur broad, femur with short setae anteriorly; postfemur, tibia and tarsus cylindrical with short setae, tarsus longest; no tarsal claw. Legpair 2 not modified, penes inconspicuous at base.

Leg 7 coxa not elongated. Prefemoral pads beginning leg 4, on midbody legs ca 1 1/3× as long as prefemur. Midbody legs about as long as ring diameter; podomere lengths (coxa+trochanter= prefemur)>tarsus>(femur=postfemur=tibia); claw ca 1/4 tarsus length.

Anterior gonopods (Figs 5, 6A) with massive coxa (**c**) laterally shouldered at half height, projecting distally as thin process, medially with flat, anteromedially directed spine (**sp**) and laterally with apex (**lam**) wide, lamellar, marginally rounded, bent medially and bearing minute, irregular projections with pointed edges on anterior surface, and with anteriorly directed tab at base of lamella (**at**). No pseudoflagellum. Flagellum (**fl**) curving smoothly behind telopodite, reaching ca 1/2 gonopod height, tip bifurcate with smooth, pointed lateral branch and flattened, roughened medial branch. Telopodite (**t**) slender, slightly tapered, extending a little further than coxal process; the tip bent anteriorly, divided into two blunt projections with a few closely appressed setae.

**Figure 5.**
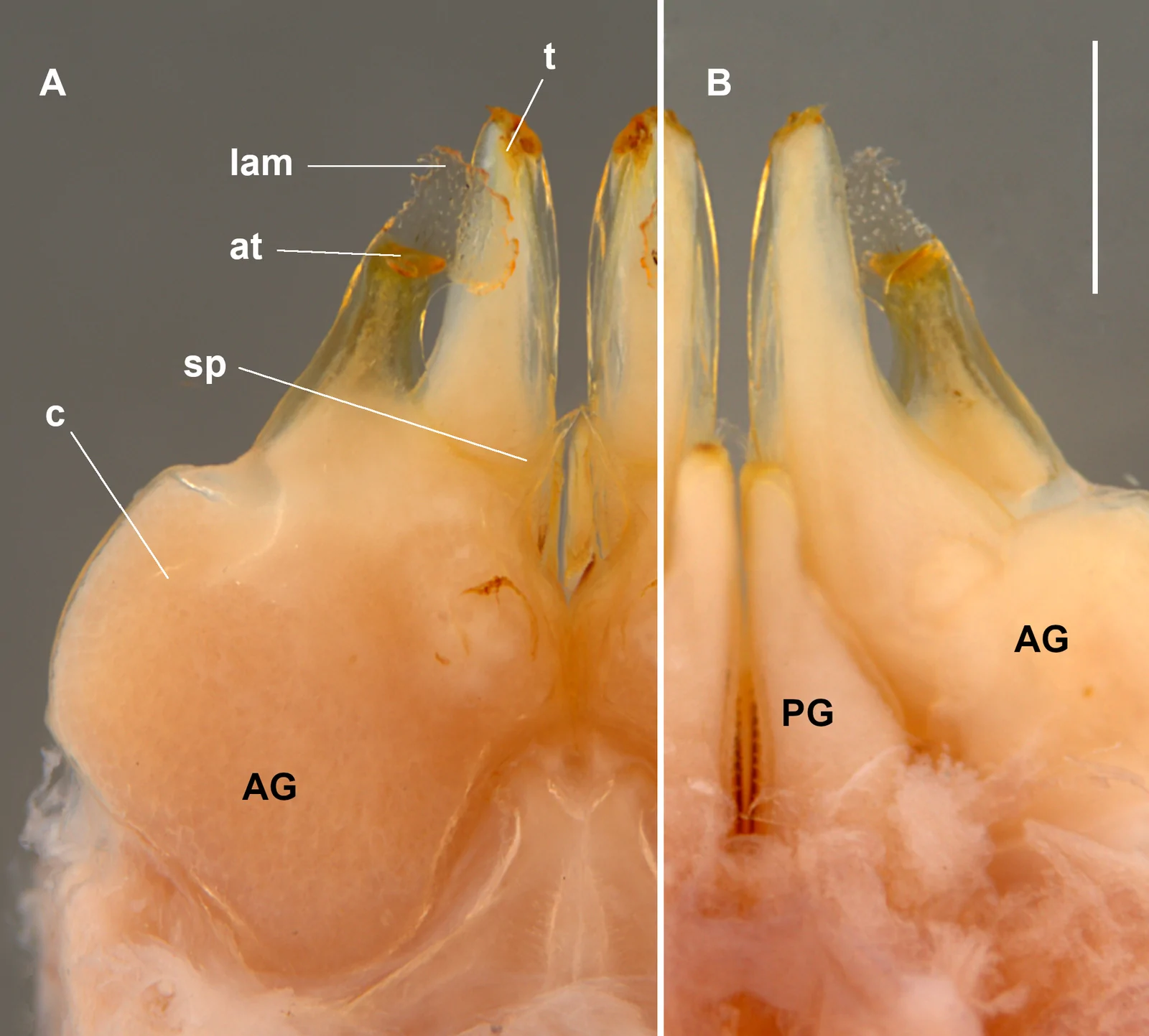
*Notonesocambala adoceta* gen. nov., sp. nov., male paratype, SAMA OM-2221. Gonopod complex in anterior (**A**) and posterior (**B**) view. Abbreviations: **AG** anterior gonopod, **at** anterior tab, **c** coxa, **lam** apical lamella, **PG** posterior gonopod, **sp** anteromedial spine, **t** telopodite. Scale bar: 0.5 mm.

**Figure 6.**
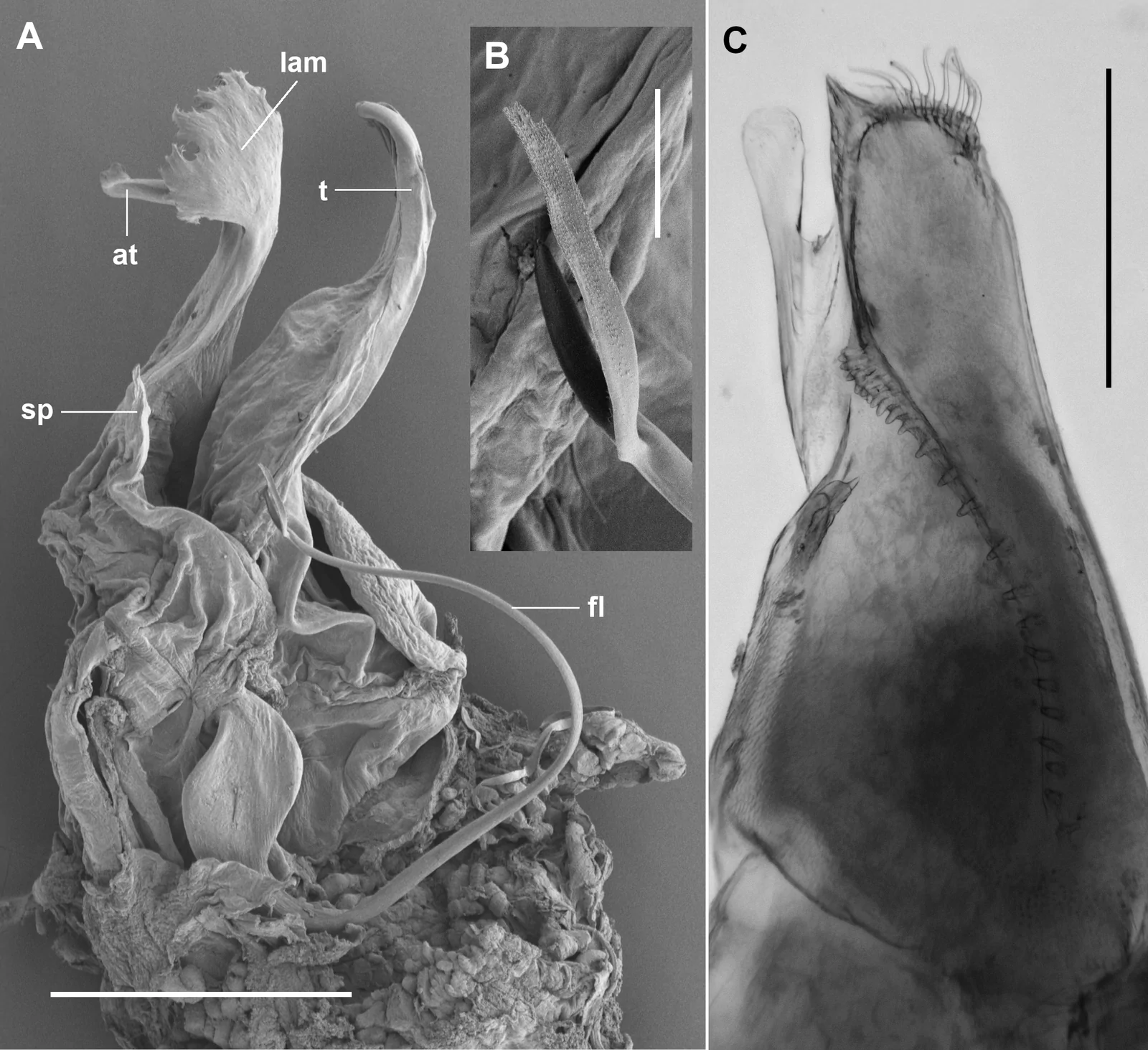
*Notonesocambala adoceta* gen. nov., sp. nov., male paratype, SAMA OM-2221. **A**. Medial view of left anterior gonopod, distorted by drying; abbreviations: **at** anterior tab, **fl** flagellum, **lam** apical lamella, **sp** anteromedial spine, **t** telopodite; **B**. Tip of flagellum, detail; **C**. Medial view of left posterior gonopod. Scale bars: 0.5 mm (**A**); 0.005 mm (**B**); 0.25 mm (**C**).

Posterior gonopods (Figs 5B, 6C) reaching to level of medial spine on anterior gonopods, laterally close to anterior gonopod coxae. Base subcylindrical, tapering distally; thin anterolateral process arising at ca 1/2 telopodite height, flattened mediolaterally, narrowing with apex rounded and slightly expanded. Telopodite narrowing to broadly rounded apex with 7 short setae; medial surface with oblique row of ca 25 peg-like setae, the row doubled at anterior, distal end; lateral surface with thin ridge parallel and posterior to alignment with anterior gonopod coxa. At ca 1/2 telopodite height, a short distomedially directed process on anterior surface with peg-like seta at apex.

Female unknown.

##### Name.

Adjective, from Greek *adoketos* (unexpected). I collected the holotype and two paratype males in well-rotted leaf litter at the base of a eucalypt tree close to a small stream. This microhabitat and the large size of the live animals both suggested to me that the specimens were in Iulomorphidae, and I was surprised to find on dissection that they were cambalids.

##### Distribution.

Known from two sites ca 10 km apart in the northwestern portion of Kangaroo Island, South Australia (Fig. 1C).

##### Remarks.

This species is longer, more slender and darker in live colouring than undescribed Cambalidae occurring elsewhere on Kangaroo Island (specimens in SAMA), and walks quickly when disturbed rather than curling up and freezing.

## Discussion and conclusions

Mean annual rainfall data in gridded, modelled values are available for 1976–2005 on the South Australian government’s spatial data website, “NatureMaps” (http://spatialwebapps.environment.sa.gov.au/naturemaps/, accessed 2 September 2026). The northwestern portion of Kangaroo Island is considerably wetter than the lower Yorke Peninsula, being modelled at ca 600–800 mm/yr vs ca 450 mm/yr near Marion Bay, and the modelled mean annual rainfall at the type locality for *C. deckeri* gen. nov., sp. nov. is ca 810 mm. If the species still occurs on the much drier lower Yorke Peninsula, it may be living in voids in the limestone that outcrops extensively in the area. On Kangaroo Island the species has been found on flat, slow-draining land and in stream gullies, in both cases with native woodland cover.

*Colobothrix deckeri* gen. nov., sp. nov. may once have been more widely distributed in the wetter, western half of Kangaroo Island. Agricultural clearing and frequent bushfires (Bauer 1959, pp. 207–210; Bonney et al. 2020) have removed or degraded much of the original native woodland over the past two centuries, and the millipede fauna in native vegetation remnants on and near farms is now overwhelmingly dominated by introduced European Julidae (personal observations).

Currently the known Kangaroo Island localities for *C. deckeri* gen. nov., sp. nov. are in Flinders Chase National Park (type locality), in the Western River Wilderness Area (2010 site) and on private property adjoining Snug Cove Road. The 2010 Western River site is also the type locality for *Notonesocambala adoceta* gen. nov., sp. nov., whose one other known locality is on private property.

The northwestern portion of Kangaroo Island is home to another rare invertebrate, the assassin spider *Zephyrarchea austini* Rix & Harvey, 2012. *Z. austini* was described from a single site (the same as the 2010 site for *C. deckeri* gen. nov., sp. nov. and the 2026 type locality for *N. adoceta* gen. nov., sp. nov.) and was feared to be threatened by fire, vegetation dieback and climate change (Rix and Harvey 2012). A targeted survey by Jessica Marsh following the 2020 Kangaroo Island bushfires recovered two additional specimens of *Z. austini* on private property ca 4 km west of the type locality (Marsh and Glatz 2022, pp. 487 and 492), with *C. deckeri* gen. nov., sp. nov., *N. adoceta* gen. nov., sp. nov. and other litter invertebrates as by-catch in the survey.

To judge from recent collections and SAMA specimens, the most widespread native polydesmidan species in native vegetation over much of Kangaroo Island is the paradoxosomatid *Somethus inflatus* (Jeekel, 2002), which is also found on the nearby Fleurieu Peninsula on the mainland (Decker 2016). Although most of the Fleurieu Peninsula has been cleared for agriculture, dalodesmids may still occur in forest remnants and plantations in the wetter, central parts of the peninsula, and in deep gullies in Deep Creek Conservation Park on the peninsula’s southern coast.

## Supplementary Material

XML Treatment for Colobothrix

XML Treatment for Colobothrix deckeri

XML Treatment for Notonesocambala

XML Treatment for Notonesocambala adoceta

## Additional information

### Artificial Intelligence (AI) use

The authors accept full responsibility for the content of the manuscript, including the disclosure of any use of AI.

No AI tools were used in the preparation of this manuscript.

### Author ORCIDs

R. Mesibov https://orcid.org/0000-0003-3466-5038

### Data availability

All of the data that support the findings of this study are available in the main text.
